# Representation of Global and National Conservation Priorities by Colombia's Protected Area Network

**DOI:** 10.1371/journal.pone.0013210

**Published:** 2010-10-12

**Authors:** German Forero-Medina, Lucas Joppa

**Affiliations:** 1 Nicholas School of the Environment, Duke University, Durham, North Carolina, United States of America; 2 Microsoft Research, Cambridge, United Kingdom; Stanford University, United States of America

## Abstract

**Background:**

How do national-level actions overlap with global priorities for conservation? Answering this question is especially important in countries with high and unique biological diversity like Colombia. Global biodiversity schemes provide conservation guidance at a large scale, while national governments gazette land for protection based on a combination of criteria at regional or local scales. Information on how a protected area network represents global and national conservation priorities is crucial for finding gaps in coverage and for future expansion of the system.

**Methodology/Principal Findings:**

We evaluated the agreement of Colombia's protected area network with global conservation priorities, and the extent to which the network reflects the country's biomes, species richness, and common environmental and physical conditions. We used this information to identify priority biomes for conservation. We find the dominant strategy in Colombia has been a proactive one, allocating the highest proportion of protected land on intact, difficult to access and species rich areas like the Amazon. Threatened and unique areas are disproportionately absent from Colombia's protected lands. We highlight six biomes in Colombia as conservation priorities that should be considered in any future expansion of Colombia's protected area network. Two of these biomes have less than 3% of their area protected and more than 70% of their area transformed for human use. One has less than 3% protected and high numbers of threatened vertebrates. Three biomes fall in both categories.

**Conclusions:**

Expansion of Colombia's Protected Area Network should consider the current representativeness of the network. We indicate six priority biomes that can contribute to improving the representation of threatened species and biomes in Colombia.

## Introduction

Human activities have transformed Colombia's natural landscapes, mainly through cattle ranching and agriculture [1], [2], [3]. Some ecosystems, like montane tropical forests, currently occupy less than 30% of their original extent [4], [5]. Starting in the 1960s Colombia began to build a network of protected areas in order to repel these land cover changes and protect biodiversity. This network now covers more than 10% of the country's territory, although some biologically unique areas remain under-protected and face serious threats. Colombia possesses extraordinary biological diversity. Among countries, it harbors the highest number of known bird species, and is second for known plants and amphibians [6]. Colombia has the potential to preserve a considerable portion of the world's biodiversity, making its conservation schemes both regionally and globally relevant.

Because conservation resources are limited, scientists and organizations have proposed different global prioritization schemes to maximize conservation investment [7], [8]. Global priorities differ in their approaches. Some of them prioritize highly vulnerable areas, a strategy defined as a reactive, while others concentrate on less vulnerable, well-preserved areas, following a proactive strategy [8]. We ask: to what extent do these global schemes overlap with finer scale national protection targets? Evaluating how a country's network fits into global conservation priorities allows us to understand if any of these priorities are over or under represented and helps to identify the strategy decision makers followed in the creation of the network. At the national level, increasing evidence shows that protected areas are often non-randomly located. Protected areas are often on steep slopes, high elevations, poor soils, and other places unrepresentative of the common climatic, geographic, or biotic conditions of the country [9], [10], [11], [12].

We analyze Colombia's network of protected areas to understand how the network agrees with global prioritization schemes and to what degree it represents the biotic and abiotic conditions of the country. This is important to do as protected area networks are the most important global strategy for biodiversity conservation, and are the first line of defense in efforts to slow habitat degradation and prevent species extinctions [13], [14]. The term “protected area” is really a mix of different legal designations [15], but most are used in one way or another to help carry on the main function of preserving vulnerable/unique sites, for the maintenance of species, evolutionary history, ecosystems, or ecosystem services.

We conduct a comprehensive assessment of Colombia's protected areas network in two ways. We start from a global perspective by determining how protected areas in the country fit into commonly accepted global conservation priorities. Then, we ask if the network properly represents the biomes, species richness, threatened vertebrate species, and common environmental and physical conditions within the country. These two questions address independent decision-making criteria. Additionally, we identify six priority biomes for conservation based on two criteria. In the first, we consider low protection, based on the percent of the biome's extension protected by the network, and high land use change. In the second, we determine biomes with low protection and high levels of threatened species. Protecting Colombia's abundant biodiversity should be a conservation matter of global importance, and our approach contributes to identifying potential directions for the selection of conservation priorities in Colombia.

## Methods

### Study Area

Colombia is located in northwestern South America, and has an area of 1,142,00 km^2^. The climate is predominantly tropical with temperature affected by altitude in the Andes mountain range, which subdivides into three branches when it enters the country. Mountain position and elevation gradients contribute to the presence of a variety of climatic conditions. These conditions are represented by 34 different continental and marine biomes and 314 ecosystem types, as recognized by National Institutions [16]. For the present analyses we considered Protected Areas in IUCN categories I to VI, which include areas from the Colombia Natural National Parks System (categories I to IV) and National Protective Forest Reserves (category VI). We also considered Regional Protected Areas in our analysis of representativeness. These Regional protected areas do not have an IUCN category because their level of protection can vary from one place to another. However, their number is increasing in Colombia, and they can play an important role in conservation. The data used corresponds to the most up to date (2009) spatial information on Colombia's Protected Areas [17], comprising 105 National protected areas, and 219 Regional protected areas.

### Global Conservation Priorities

To understand how protected areas in Colombia are representative of global conservation priorities, we estimated the number of individual protected areas and the percentage of the total land protected located within each of seven recognized global conservation priorities. These correspond to prioritization templates published over the past decade by various organizations. Brooks et al. [8] reviewed their methods and impacts. They are: Frontier Forests [18], Last of the Wild [19] and Wilderness Areas [20], which follow a proactive approach; Biodiversity Hotspots [13] and Crisis Ecoregions (updated version, Hoekstra personal communication), which follow a reactive approach; Endemic Bird Areas (EBAs) [21] and Centers of Plant Diversity [22], which do not incorporate vulnerability but only a uniqueness criterion [8].

We also estimated the percent of the G200 regions [23] in Colombia that is protected by the network and the number of sites identified by the AZE (Alliance for Zero Extinction) that are within protected areas. The G200 ecoregions are conservation priorities aimed at protecting representative examples of all of the world's ecosystems. They are also areas with exceptional concentrations of species and endemics [24]. The AZE is a global initiative that seeks to prevent extinctions by identifying and safeguarding key sites where species are in imminent danger of disappearing [25].

### Representation of local biomes

For determining the representation of national priorities, we first estimated the proportion of each biome's total area that is protected. Biome types follow the classification from the most recent version of the map of Colombian Ecosystems (Figure S1) [16]. The insular biomes from the Caribbean and the Pacific were not included on these analyses. We first considered the protection under National protected areas and then estimated the protection under both National and Regional protected areas together. In this way we could examine how and where regional protected areas are complementing the protection by national protected areas.

### Representation of biophysical variables

We analyzed the distribution of protected areas across Colombia relative to elevation [26], slope (derived from elevation data), species richness (amphibians, mammals, and breeding birds), agricultural suitability [27], distance to roads [28], and distance to urban areas [29]. We inverted the original agricultural suitability index so that it would indicate increasing suitability and be more intuitive. All of the above datasets were in raster (grid) format. We used ArcGIS 9.3 to harmonize projections, cell size (1 km^2^), and extent. We carried out all further analyses in the program R (version 2.8.1) [30].

We first binned each of the variables into discreet intervals (elevation: 100 m, slope: 1°, richness: 50 species, distance to roads: 5 km, distance to urban areas: 5 km, agricultural suitability: 1–8 increasing suitability index). For each of these variables we plotted the difference between the percent of Colombia's terrestrial land surface, and the percent of Colombia's protected area network at each interval. Doing this highlights the areas where Colombia's protected lands differ from what we would anticipate given the distribution of each variable across the country. Numbers of vertebrate species in each biome were extracted from richness maps compiled by Jenkins [31] from the Global Amphibian Assessment, the Global Mammal Assessment [6] and NatureServe, version 3.0 of the Birds of the Western Hemisphere [32].

### National Priority Biomes for Conservation

We established national priority biomes for conservation in Colombia using level of protection, degree of land cover transformation, and numbers of threatened species. To be a priority biome first required protection levels below 3%. Additionally, a biome must either have more than 70% of its natural land cover transformed by human activities [16], contain more than 12 threatened vertebrate species, or both. The cutoff for the number of threatened species corresponds to >50% of the maximum number for a single biome. The combination of protection level and land cover transformation is an approach similar to the one used for global crisis ecoregions [33]. The rationale is that areas that are experiencing high levels of land cover transformation and have low protection require immediate attention. Protecting these areas will contribute to preserving the diversity of biomes in the country, along with their characteristic fauna and flora. Additionally, extinction is irreversible, so protecting biomes with high concentrations of threatened species is a priority. We identify biomes that meet all three criteria as top national-level priorities.

## Results

### Global priorities

**Figure 1:**
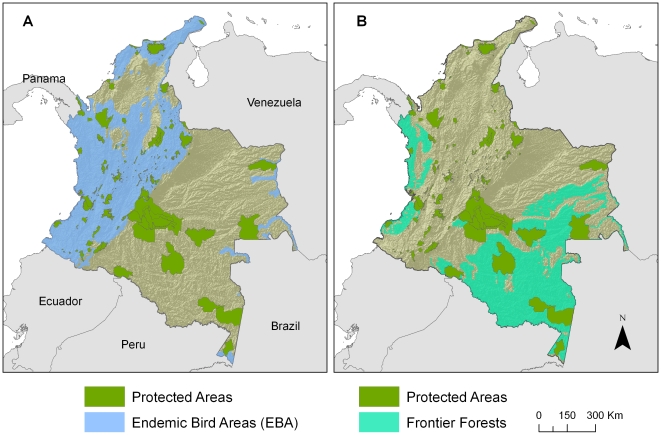
Best represented global conservation priorities in Colombia. A) Endemic Bird Areas are the Global Biodiversity Conservation template with the highest number of individual protected areas (National and Regional) within Colombia; B) Frontier Forests (proactive approach) are the template with the highest percent of the total protected area in the country.

The highest numbers of individual protected areas are located in Endemic Bird Areas and Biodiversity Hotspots. Both of these are global priorities because of their high number of endemic species or high vulnerability (Table 1). However, the highest proportion of protected land in Colombia is located within Frontier Forests, Last of the Wild and Wilderness Areas (Figure 1). These are all well-preserved, isolated, and low vulnerability regions. This pattern remains when considering only National protected areas or both National and Regional protected areas together (Table 1). When the Regional protected areas are included in the analysis, the proportional area protected for EBAs, Hotspots, and Crisis Ecoregions increases. However, they remain less protected than areas of low vulnerability. Six of the twelve G200 terrestrial ecoregions have more than 10% of their area protected, while two of them have no protection at all. The Alliance for Zero Extinction (AZE) has identified 48 important places in Colombia so far, of which only 15 (31%) are represented by the network.

**Table 1:** Representation of Global Conservation Priorities in Colombia's Protected Area Network.

Global Conservation Template	Number of PAs in IUCN category		Total Number	% Total Protected Area (National PAs)	% Total Protected Area (National + Regional PAs)
	I-IV	V-VI			
Frontier Forests (P)	17	4	21	68.7	70.0
Last of the Wild (P)	12	3	15	66.6	68.0
Wilderness Areas (P)	12	2	14	62.1	64.7
Centers of Plant Diversity*	16	9	25	55.1	41.8
Endemic Bird Areas (EBA)*	42	50	92	54.5	62.9
Hotspots (R)	36	53	89	43.9	58.1
Crisis Ecoregions (R)	31	41	72	39.3	53.6

Number of National Protected Areas (PAs) and proportion of the total protected land in Colombia (both for National and combined PAs) located within different global conservation priorities. Type of strategy according to Brooks et al. (2006): P =  Proactive; R =  Reactive;

*Does not consider vulnerability.

### Representation of Colombian biomes by the network

**Figure 2:**
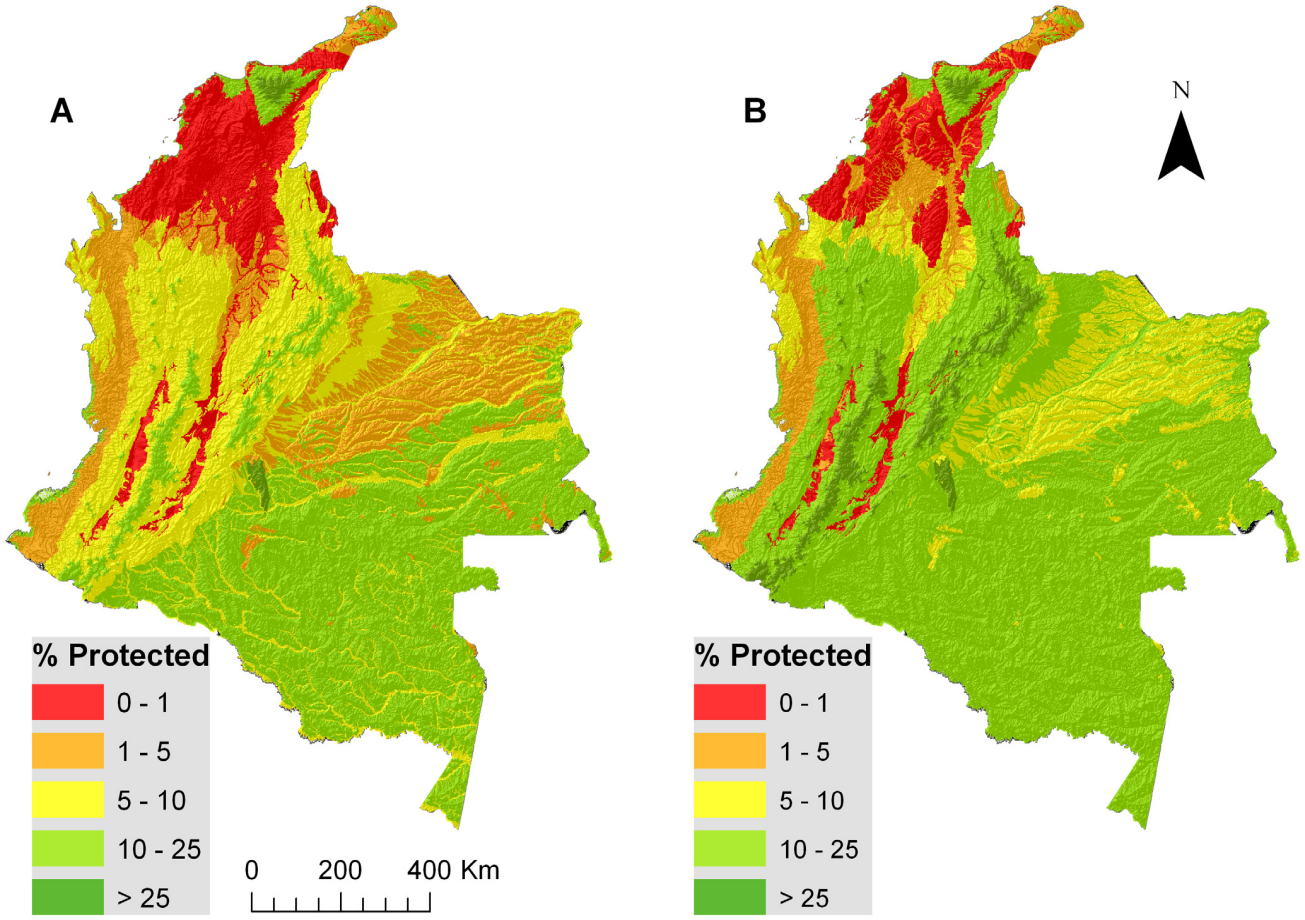
Percent of each biome's area protected by different networks. A) National network (IUCN I-VI) and B) National and Regional Networks together.

When considering only National protected areas, ∼30% of Colombia's biomes have at least 10% of their area protected, although nearly 70% have at least some degree of protection. When considering National and Regional protected areas together, these percents increase to ∼40% and ∼90% respectively (Table 2). The biomes with best coverage are the Orobioma Alto de Santa Marta and Orobioma de la Macarena. Nonetheless, 11 biomes are entirely absent from the National protected areas network. This number decreases to three by considering National and Regional protected areas together (Table 2). The biomes with less than 10% protection are located in the Pacific and Caribbean regions, the Cauca and Magdalena River Valleys and lower Andes, and part of the Orinoquia (Figure 2). Although considering National and Regional networks together in the analysis improves the protection level for some areas, (ex: Orinoquia, lower Andes, and lower Magdalena River valleys), other regions remain with the same low levels of protection. These are the Pacific region, the upper Magdalena and Cauca River valleys, and the Caribbean (Figure 2).

**Table 2:** Percent of area protected for the Colombian Biomes.

Biome	Area (km^2^)	IUCN	IUCN	Including
		I -IV	I - VI	Regional PAs
Helobioma de La Guajira	905.10	0.0	0.0	0.0
Orobioma de San Lucas	8573.55	0.0	0.0	0.0
Orobiomas azonales del Valle del Patφa	1242.99	0.0	0.0	0.0
Helobiomas del Río Zulia	132.48	0.0	0.0	0.1
Helobiomas andinos	333.51	0.0	0.0	0.2
Zonobioma alternohígrico y/o subxerofítico tropical del Alto Magdalena	10279.62	0.0	0.0	0.4
Zonobioma seco tropical del Caribe	55591.36	0.2	0.3	0.5
Zonobioma alternohígrico y/o subxerofítico tropical del Valle del Cauca	5453.52	0.0	0.0	0.5
Orobiomas azonales de C·cuta	1102.38	0.0	0.0	0.6
Zonobioma húmedo tropical del Catatumbo	2553.31	0.9	0.9	1.1
Zonobioma del desierto tropical de La Guajira y Santa Marta	6677.61	1.2	1.2	1.2
Zonobioma húmedo tropical del Pacífico y Atrato	34314.71	0.8	1.9	2.7
Helobiomas del Valle del Cauca	1401.64	0.0	0.0	2.8
Helobiomas del Magdalena y Caribe	33300.47	0.2	0.2	2.9
Helobiomas del Pacífico y Atrato	12761.41	3.0	3.4	3.4
Orobiomas azonales Río Dagua	59.65	0.0	0.0	3.9
Zonobioma h·medo tropical del Magdalena y Caribe	33999.28	3.6	4.5	5.8
Peinobiomas de la Amazonia y Orinoquia	121602.69	4.0	4.0	7.4
Orobioma del Baudó y Darién	12883.16	6.6	10.0	10.0
Helobiomas de la Amazonia y Orinoquia	116671.84	6.8	6.9	11.5
Halobiomas del Pacífico	5036.88	10.1	11.3	11.6
Orobiomas bajos de los Andes	143152.53	7.6	8.4	12.7
Orobiomas medios de los Andes	75697.39	7.0	8.6	13.0
Zonobioma húmedo tropical de la Amazonia y Orinoquia	321131.22	11.7	11.7	15.9
Orobioma bajo de Santa Marta y Macuira	9944.97	17.1	17.1	17.4
Halobioma del Caribe	3984.63	9.4	16.0	19.9
Litobiomas de la Amazonia y Orinoquia	72549.33	24.1	24.4	24.8
Orobiomas altos de los Andes	41834.91	22.2	24.8	30.1
Orobiomas azonales del Río Sogamoso	443.26	0.0	0.0	33.1
Orobioma medio de Santa Marta	1741.49	63.0	63.0	63.0
Orobioma alto de Santa Marta	1576.21	92.9	92.9	92.9
Orobioma de La Macarena	2994.86	77.2	77.2	99.7
Total	1139927.96			

Percent protected under National protected areas is discriminated by IUCN categories.

### Representation of biophysical conditions

**Figure 3:**
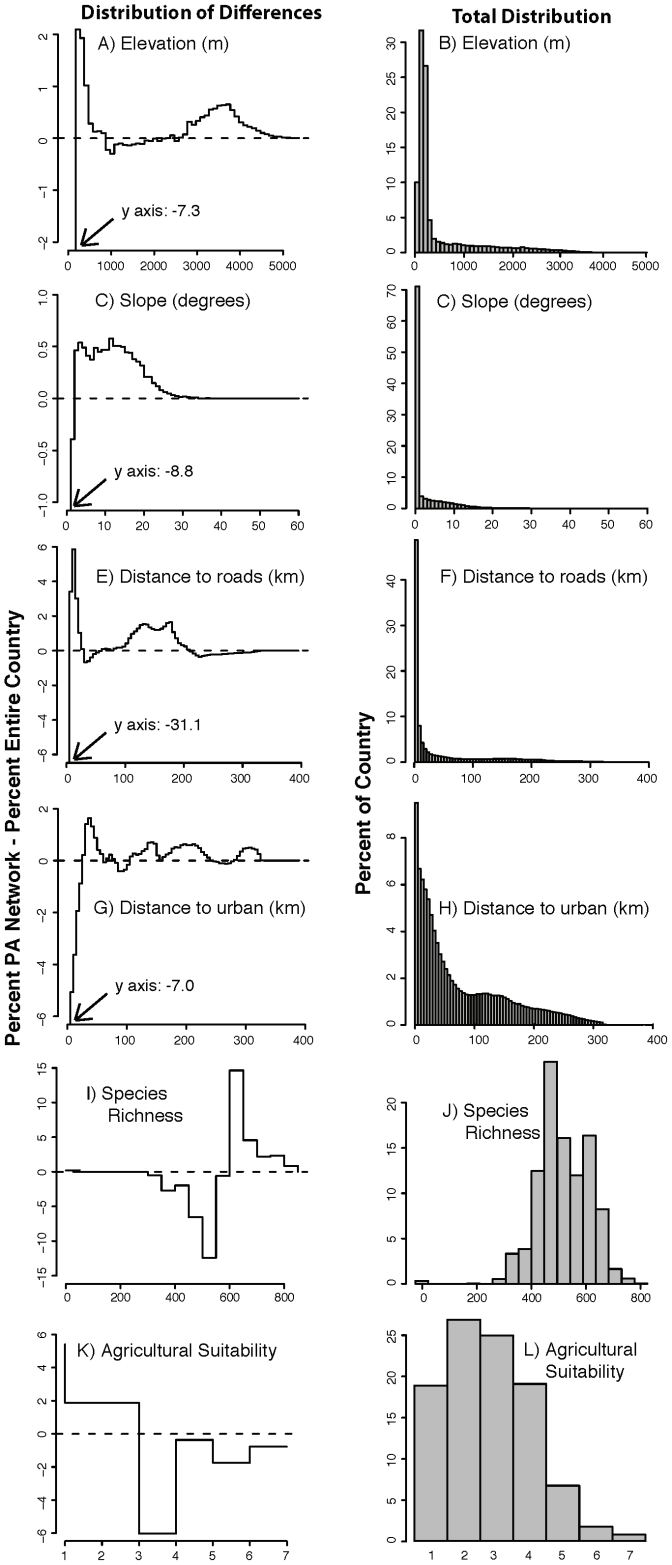
Distribution of Biophysical variables in Colombia's Protected Area Network. The distribution of **A**,**B**) elevation (m), **C**,**D**) slope (degrees), **E**,**F**) distance to roads (km), **G**,**H**) distance to urban areas (km), **I**,**J**) species richness, and **K**,**L**) agricultural suitability (increasing suitability from 1 to 7) across Colombia and Colombia's protected area network is shown. **Left Column**: the difference between the percent of Colombia's protected area network at each increment and the percent of the entire country within that increment. Anything above the dashed horizontal line indicates disproportionate presence of that landscape type within the protected network, while below the line is disproportionate absence. Because of the extreme negative values in several of the graphs, some of the axes in the left hand graphs have been truncated. In these cases the arrows indicate the minimum y-axis value and the maximum x-axis value. **Right Column**: the percent distribution of each variable across all of Colombia, which we provide as context for the matching graph in the left column. The axes on the right hand graphs were not truncated in order to display the full distribution of the variable.

**Figure 4:**
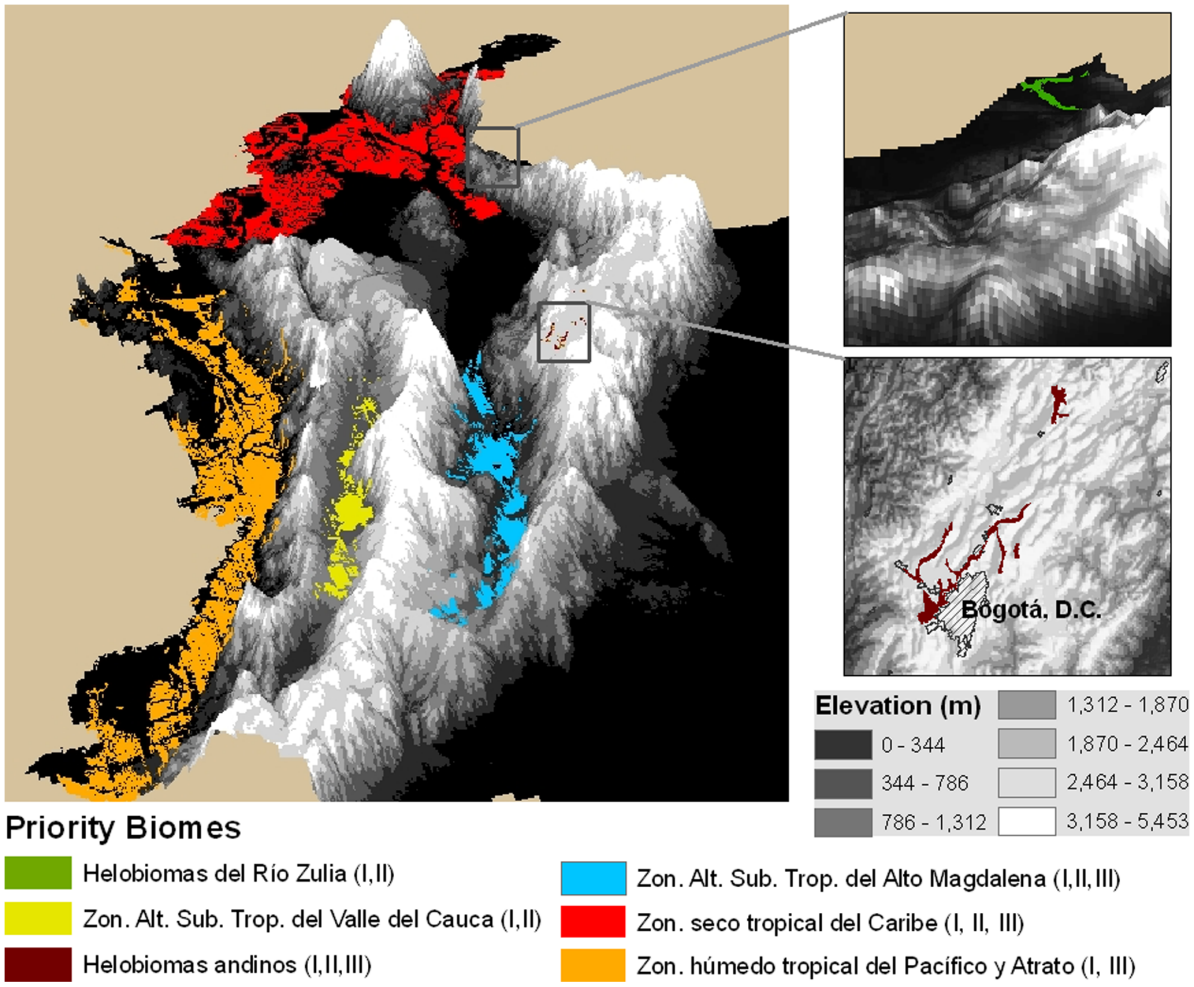
Priority Biomes for Conservation in Colombia. The six biomes identified as important for conservation using three criteria: I) low protection level (<3%), II) high land cover transformation by human activities (>70%), and III) high number of threatened vertebrate species (>12 species). The criteria met by each biome is indicated in the legend in parenthesis.

The distribution of biophysical variables indicates that the protected areas network has proportionally more area of high species richness than one would expect by chance alone (Figure 3 i,j). This is indicated by the fact that the percent of area within protected areas with richness values above 600 is higher than the proportion of the country with this same richness values (Figure 3i,j). The network is also far from highways and urban areas (Figure 3e,f and Figure 4g,h, respectively), at high elevations (Figure 3a,b) and on steep terrain (Figure 3a,b). Combined, these results dictate the network is on lands of low agricultural suitability (Figure 3k,l). For example, lowland areas below 200 m are proportionally underrepresented, while areas over 2800 m are proportionally more protected (Figure 3a,b). Excluding species richness, across all of the variables the extreme negative values (i.e, those places where protected areas are the most proportionately absent) occur on the lowest, flattest, lands that are the closest to roads and urban areas and are highly suited for agriculture.

### Priority Biomes

Using the criteria of protection, land cover conversion, and threatened species, we identified six Colombian biomes as priority regions for conservation (Figure 4). Two are priorities based only on protection and land cover conversion, one is a priority based only on protection and numbers of threatened species, and three are priorities due to all three criteria (Figure 4). All six biomes are located within Hotspots, Endemic Bird Areas, or both. Here we describe the main characteristics of these biomes, and the processes leading to their threatened status.

Two of Colombia's biomes have less than 3% of their area protected, are located in areas of dense population settlements and high road densities and hence have more than 70% of their area transformed to non-natural landscapes [16]. These biomes are the Helobiomas del Rio Zulia and the Zonobioma alternohígrico y/o subxerofítico tropical del Valle del Cauca, (Figure 4).

#### Helobiomas del Rio Zulia

This biome consists of wetlands that are under the influence of the Zulia River, along the frontier with Venezuela. Agriculture has intensively transformed the wetland vegetation in the region. Only around 10% of the original vegetation remains and only a small fraction (0.1%) is protected under Regional protected areas.

#### Zonobioma alternohígrico y/o subxerofítico tropical Valle del Cauca

Corresponds to tropical dry forests located in the upper Cauca River valley. The area has been highly transformed since the 1950's for sugar cane plantations [34]. Less than 10% of the vegetation remains, and the forest remnants correspond to secondary and highly altered vegetation [34].

One priority biome has low levels of protection, high numbers of threatened species (19 species), yet retains largely undisturbed natural land cover.

#### Zonobioma húmedo tropical del Pacífico y Atrato

**Figure 5:**
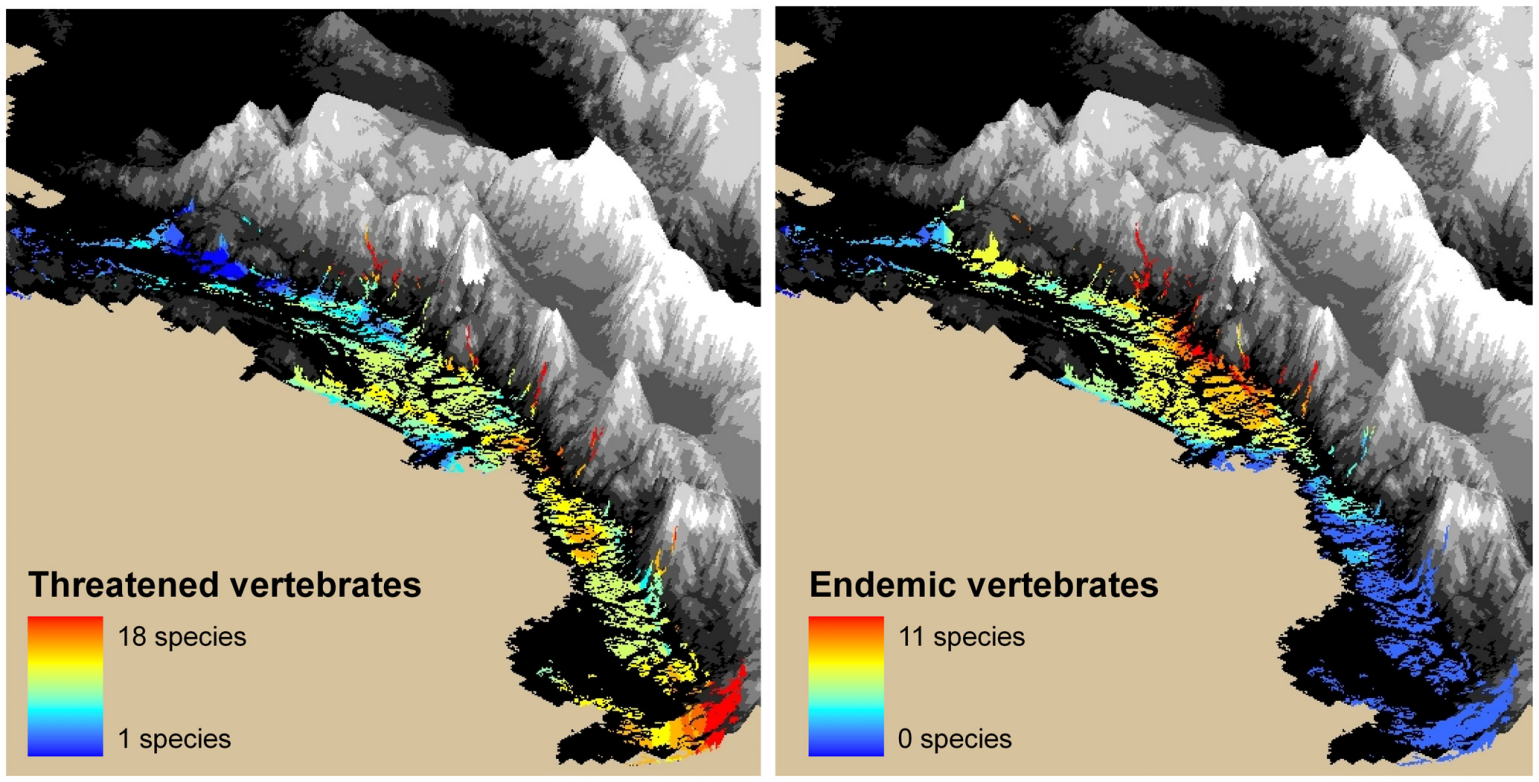
The Pacific biome *Zonobioma humedo tropical del Pacifico y el Atrato*. The number of threatened (left) and endemic (right) vertebrates is shown for the areas of the biome with remaining natural vegetation.

This tropical rain forest retains considerable portions of natural vegetation, which represent the best-preserved part of the Tumbes-Choco-Magdalena hotspot (Figure 5). It is an area of high endemism, containing some of the richest tropical moist forests on earth. The highest concentration of endemics occurs close to the eastern boundary of the biome, close to the western Colombian Andes (Figure 5). This biome also presents a high concentration of threatened species. Many of these have restricted ranges, like the Colourful Puffleg (*Eriocnemis mirabilis*), with a known range of only 31 km^2^ [35]. In other cases, the Pacific region represents the only part of a threatened species range in South America, like Baird's Tapir (*Tapirus bairdii*). Most of the threatened species occur on the southern part of the biome (Figure 5). Unfortunately, this is where most colonization, road building, and development projects have taken place. Although this region retains some extensive natural forests, many less detectable threats are taking place. Activities such as illegal logging and mining for gold and platinum are common. In addition, there are development projects either approved or to be approved. These include hydrocarbon exploration and new roads that would go through well-preserved areas [36], [37], [38].

Three of our six priority biomes fall into all three of our criteria, with low protection levels, high land cover conversion, and high numbers of threatened species. We mark these as the top conservation priorities in Colombia. The biomes are the Zonobioma alternohígrico y/o subxerofítico tropical del Alto Magdalena (16 species), Helobiomas Andinos (15 species), and the Zonobioma seco tropical del Caribe (13 species) (Figure 4).

#### Helobiomas Andinos

It corresponds to the wetlands of Cundinamarca and Boyacá. The biome is a system of swamps and lakes that used to cover a considerable portion of the plateau where the capital city of Bogotá is located today. This biome contains endemic species of vertebrates, and unique assemblies of plant communities [39]. These wetlands are also important areas for migratory birds from North America [40]. The high population density in the area contributed to the transformation of land for agriculture, cattle farming and urbanization [16], [41]. Human activities have transformed more than 90% of the land and only a very small fraction (0.2%) is protected under Regional protected areas.

#### Zonobioma alternohígrico y/o subxerofítico tropical del Alto Magdalena

This biome has warm temperatures and a marked dry season, when plants experience water deficit [42]. It is included within the broad biome of Tropical Dry Forest [16], and is located in the upper basin of the Magdalena River. This area is used intensely for cattle and agriculture, and is one of the leading producers of both in Colombia. Most of the forest patches remaining are the ones located on steep hills where agriculture is not viable. Therefore, remaining areas with vegetation should be protected, and reforestation practices should be implemented to connect the smaller remnants.

#### Zonobioma seco tropical del Caribe

This biome is within the broad biome of Tropical Dry Forest [16], [42]. Extensive cattle farming and urban development have transformed its landscapes. Although human activities have severely transformed around 70% of its original vegetation, only 0.5% is protected under National and Regional protected areas.

## Discussion

**Figure 6:**
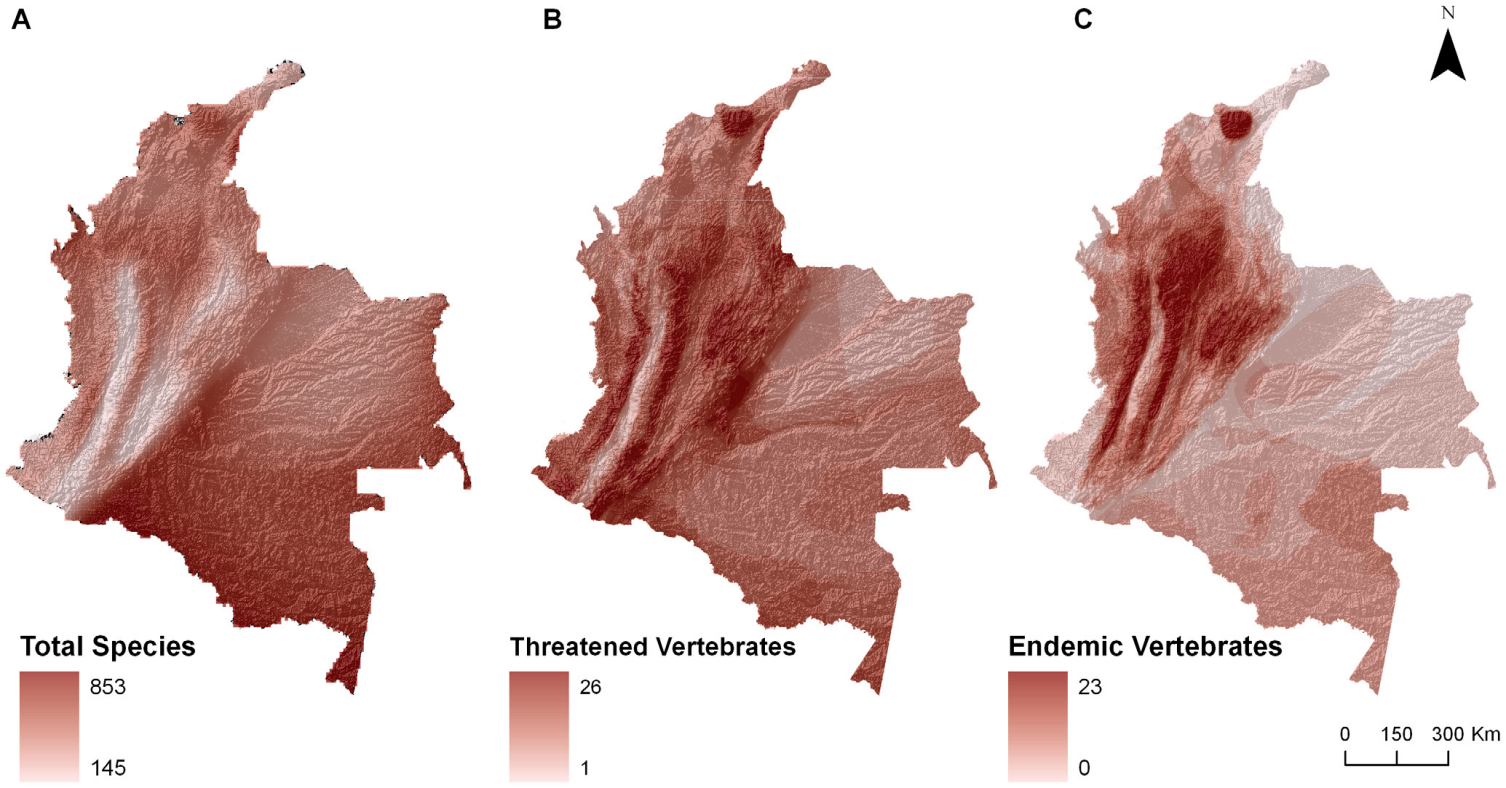
Distribution of richness, endemics, and threat for vertebrate species in Colombia. A) species richness (amphibians, mammals, birds), B) number of threatened species, and C) number of endemic species in Colombia.

Our results indicate that the dominant strategy in Colombia has been a proactive one, allocating a higher proportion of the protected land on well-preserved, remote and species rich areas, mainly in the Amazon. The smaller size of parks in more threatened areas like Hotspots may relate to the reduced availability of large portions of land for protection, or to the price of land near urban settlements. Given these constraints, the organizations that have helped in designing the National Natural Parks have clearly identified areas of high biological richness. Unfortunately, areas with high total richness do not coincide with areas that contain high numbers of threatened and endemic species (Figure 6). Threatened areas with high numbers of endemic species have low overall protection. The poor representation of Hotspots and Crisis Ecoregions in the network is a clear example of this. Local governments, however, have placed Regional protected areas preferentially in these high threat/endemic areas. This selective location is shown by the percent of the total protected area within Hotspots, Endemic Bird Areas, and Crisis Ecoregions increasing when we include Regional protected areas in the analysis. This means that Regional protected areas are complementing National protected areas. However, because of the small size of these Regional Protected Areas they do not represent a big proportion of the total protected land in the country. Overall, the combined National and Regional networks protect well-preserved, isolated areas. Protecting desirable, high-value lands can cost more than doing so in remote areas with few threats. However, the biodiversity value, ecosystem services provided by these natural areas, and potential for inclusion in new projects like REDD, can help overcome the potential high opportunity cost of conserving these lands.

While a considerable portion of Colombia's biomes have at least some degree of protection, only around 40% of them have 10% or more of their area protected. Biomes located in the Amazon are the ones with high levels of protection, while biomes on the Caribbean, the Magdalena and Cauca Valleys, part of the Pacific Region, lower Andes, and the northern Orinoquia regions have low protection. Although Regional protected areas have been created in some of these Biomes (increasing their level of protection), some other biomes remain under represented. Local governmental institutions, which have varying budgets from one region to another, create Regional protected areas. This might explain the lack of Regional protected areas complementing the National network in some Biomes where fewer resources are available. Therefore, future expansion of the National protected areas network requires considering not only the presence of Regional protected areas, but also the potential of local institutions for creating new Regional protected areas.

The patterns of distribution of Protected Areas in Colombia correspond with patterns observed at the continental and global scale. Globally, Protected Areas have a clear bias toward particular biogeographic regions and biomes [43]. In the Neotropics realm, the Tropical and Subtropical moist leaf forest outstands, having more than 30% of its area protected. This high level of protection however is due to the large proportion of land protected in the Amazon. Since 2003, most Protected Areas in this realm have been created in the Amazon [43], following a proactive approach, like Colombia.

In this analysis, we are concerned with biomes that have very low or no protection, high levels of threat, and/or many threatened species. In this case, the proportion of each biome's area transformed for human use, and the relative high density of roads indicate threat. All six biomes identified require immediate protection in order to preserve their unique biological communities. They are all located within Hotspots, on areas with high numbers of endemic species. Except for the Helobiomas Andinos they are all located in lowlands. Although at a national scale lowlands have been less transformed than Andean areas, they are experiencing higher rates of transformation and thus account for most of the land conversion in recent decades [41]. The dry tropical forest of the Caribbean, the humid tropical forests of the Magdalena and the High-Andean alluvial forests were also identified in previous studies as being most vulnerable to forest conversion in terms of the proportion of their remnant area predicted to be transformed [44].

Further analyses within the identified biomes should be conducted in order to determine the best sites for creating new protected areas. Three of the six biomes have low levels of protection, high transformation of their original extent into human land uses, and high numbers of threatened vertebrates. These are the Helobiomas Andinos, the Zonobioma alternohígrico y/o suxerofítico Tropical del Alto Magdalena, and the Zonobioma seco tropical del Caribe. We suggest these as top priorities for conservation under a reactive approach, seeking to protect vulnerable areas.

Many biomes within the Pacific coast show levels of protection between 1–5%. New protected areas that are up to the task of mitigating the current and future effects of land cover changes are required. If not, the region will have a fate similar to other hotspots where less than 10% of the original vegetation remains. This would have an enormous cost for diversity and the economy. Therefore, if new protected areas will follow a proactive approach, on remaining natural forests and remote lands, the Pacific region is a high priority.

We did not consider private reserves, indigenous reserves or collective lands inhabited by afro-Colombians in this study, and have restricted our analyses to the protected areas with IUCN categories I to VI. That said, there is increasing evidence that indigenous reserves can contribute to forest protection [45], [46], [47]. The largest indigenous reserves in Colombia are located in the southeastern part of the country, in the Amazon and Orinoquia regions, where our analyses show already considerable coverage by the protected area network. The other area with a high concentration of indigenous reserves is the Pacific Region, where most biomes have less than 5% of their area protected.

The collective titling of lands traditionally inhabited by Afro-Colombian and indigenous groups has been one of the most important legal and territorial developments in Colombia in recent decades. In the Pacific alone (∼11 million hectares) more than 5 million hectares have been titled to over 150 black communities [48], and there are more than 100 indigenous reserves that occupy some 1.2 million hectares. Together, indigenous and collective afro-Colombian territories represent more than 30% of Colombia's territory [49], [50], almost three times the proportion under Protected Areas. These lands are collectively managed. This represents a unique opportunity for the conservation of well-preserved and biologically unique areas in the country. In future work it will be important to evaluate the coverage of these indigenous reserves and collective territories of afro-Colombians, to understand how they are complementing the representation of the IUCN classified network.

The question of whether protected areas truly do mitigate environmental threats has gained increasing attention [11], [51], [52]. A major contribution of these studies has been to show the importance of location for the success of conservation investments [9], [11]. The highest proportion of protected area in Colombia is preferentially located in areas with low deforestation threats (i.e. far from roads and urban settlements, at high elevations and on steep slopes, and on less suitable land for agriculture). On the other hand, it has been shown that in Colombia deforestation is predicted to be greater in areas with fertile soils, gentle slope, near to settlements, roads and rivers [44]. Thus, it is important to consider, for the future expansion of the network, if it is better to allocate new parks in areas that present a high threat, like hotspots and crises ecoregions. Protected areas within remote, well-preserved regions may already be protected *de facto* by their isolation. Logic dictates that protected areas can only be effective at preventing land cover change if they are located in places that would be destroyed in the absence of protection [9]. This strategic establishment of protected areas is similar to the requirement of “additionality” in REDD projects [53]. Thus, protected area allocation might go hand-in-hand with REDD projects where such “additionality” is required. Currently no published studies have addressed this challenge for Colombia's protected network, but as environmental threats intensify doing so will become increasingly important.

Colombia's protected area network has been located, at least partly, within all of the global conservation priorities considered here, but priority areas following a proactive strategy have been the dominant ones. The network has protected species rich biomes; and sites that are located proportionally more within areas of less threat of deforestation. Three biomes emerge as priority areas for conservation according to their lack of representation, their high level of transformation by humans, and the high number of threatened species. These are the Helobiomas Andinos, the Zonobioma alternohígrico y/o suxerofítico Tropical del Alto Magdalena, and the Zonobioma seco tropical del Caribe. We indicate another three biomes as priorities for conservation because of a combination of either low protection and high transformation, or low protection and high number of threatened species. The Pacific coast represents a priority area within a proactive approach because it retains considerable portions of natural vegetation but has relative low protection. Future expansion of the network should consider the results from its current representation of global and national interests and the present location of parks, in order to select sites where conservation can be maximized.

## Supporting Information

Figure S1 Terrestrial Biomes of Colombia. The 32 terrestrial biomes in Colombia, excluding the insular biomes of the Caribbean and the Pacific [16].
(2.86 MB TIF)
