# Supplementary figures and images for: Representation of Global and National Conservation Priorities by Colombia's Protected Area Network
Source: PLoS One. 2010 Oct 12;5(10):e13210. doi: 10.1371/journal.pone.0013210 (PMC2953503; doi:10.1371/journal.pone.0013210)

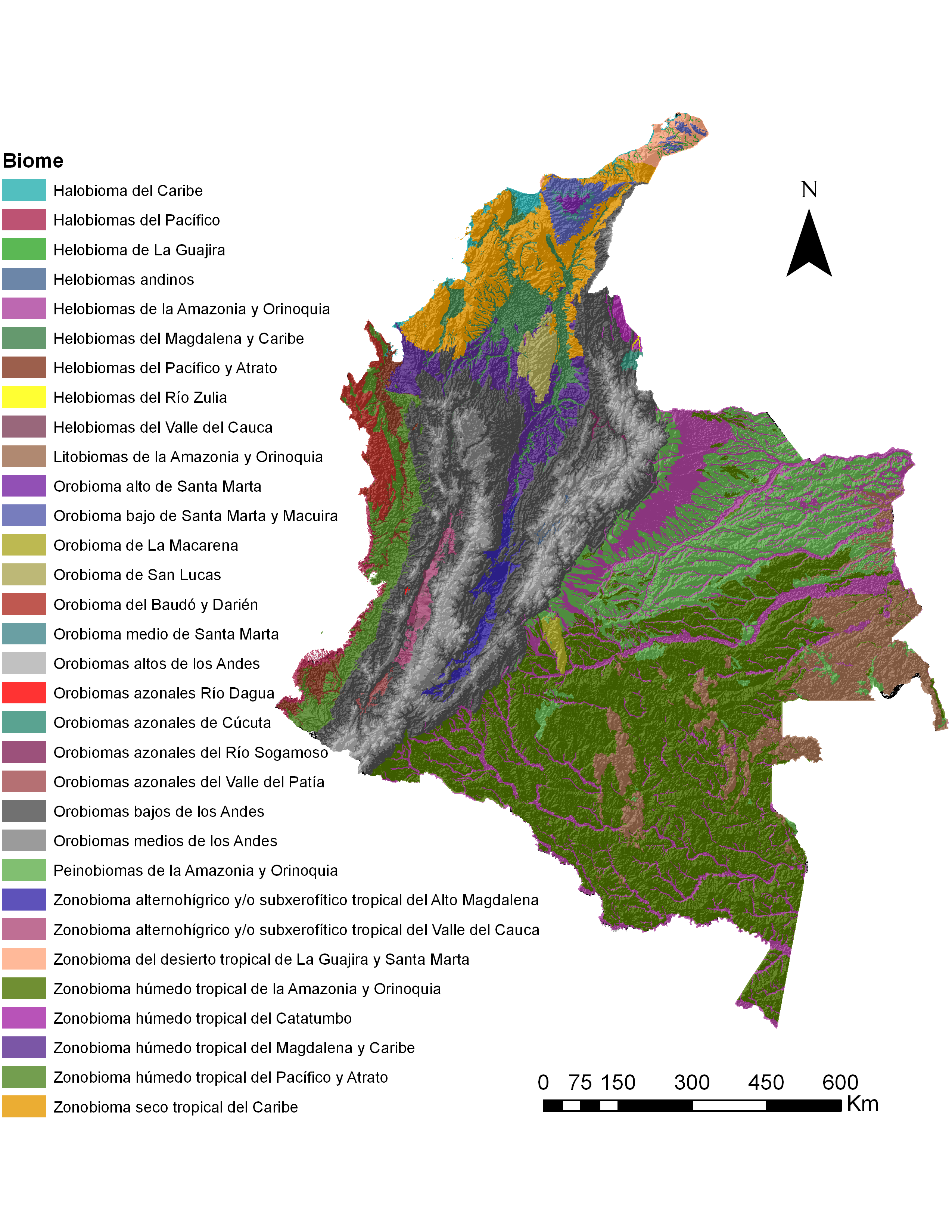

Supplement: Figure S1 — Terrestrial Biomes of Colombia. The 32 terrestrial biomes in Colombia, excluding the insular biomes of the Caribbean and the Pacific [16]. (2.86 MB TIF) [file pone.0013210.s001.tif]
